# In-depth transcriptomic analysis of human retina reveals molecular mechanisms underlying diabetic retinopathy

**DOI:** 10.1038/s41598-021-88698-3

**Published:** 2021-05-18

**Authors:** Kolja Becker, Holger Klein, Eric Simon, Coralie Viollet, Christian Haslinger, German Leparc, Christian Schultheis, Victor Chong, Markus H. Kuehn, Francesc Fernandez-Albert, Remko A. Bakker

**Affiliations:** 1 Global Computational Biology & Digital Sciences, Boehringer Ingelheim Pharma GmbH & Co. KG, Biberach an der Riß, Germany; 2 Global Computational Biology & Digital Sciences, Boehringer Ingelheim RCV GmbH & Co. KG, Vienna, Austria; 3 Translational Medicine & Clinical Pharmacology, Boehringer Ingelheim Pharma GmbH & Co. KG, Biberach an der Riß, Germany; 4 Therapeutic Area CNS Retinopathies Emerging Areas, BI International GmbH, Ingelheim, Germany; 5 Department of Ophthalmology and Visual Sciences, University of Iowa, Iowa City, IA USA; 6 Department of Veterans Affairs, Center for the Prevention and Treatment of Visual Loss, Iowa City, IA 52246 USA; 7 Global Department Cardio-Metabolic Diseases Research, Boehringer Ingelheim Pharma GmbH & Co. KG, Biberach an der Riß, Germany

**Keywords:** Computational models, Data integration, Machine learning, Cardiovascular diseases, Eye diseases, Metabolic disorders, Biomarkers, Target identification

## Abstract

Diabetic Retinopathy (DR) is among the major global causes for vision loss. With the rise in diabetes prevalence, an increase in DR incidence is expected. Current understanding of both the molecular etiology and pathways involved in the initiation and progression of DR is limited. Via RNA-Sequencing, we analyzed mRNA and miRNA expression profiles of 80 human post-mortem retinal samples from 43 patients diagnosed with various stages of DR. We found differentially expressed transcripts to be predominantly associated with late stage DR and pathways such as hippo and gap junction signaling. A multivariate regression model identified transcripts with progressive changes throughout disease stages, which in turn displayed significant overlap with sphingolipid and cGMP–PKG signaling. Combined analysis of miRNA and mRNA expression further uncovered disease-relevant miRNA/mRNA associations as potential mechanisms of post-transcriptional regulation. Finally, integrating human retinal single cell RNA-Sequencing data revealed a continuous loss of retinal ganglion cells, and Müller cell mediated changes in histidine and β-alanine signaling. While previously considered primarily a vascular disease, attention in DR has shifted to additional mechanisms and cell-types. Our findings offer an unprecedented and unbiased insight into molecular pathways and cell-specific changes in the development of DR, and provide potential avenues for future therapeutic intervention.

## Introduction

Diabetic retinopathy (DR) is a complication of diabetes that results in a progressive loss of visual acuity and is a major cause for blindness in diabetics aged 20 and older^1^. Duration of diabetes is the primary risk factor for DR, which includes prolonged exposure to chronic retinal hyperglycemia, hypertension, and hypoxia. Studies suggest three out of four diabetics develop DR within the first 20 years of disease^2^. Early stages of DR often go unnoticed until either diabetic eye-screening is performed, or the patient experiences changes in visual function and seeks medical attention. Some patients may even be unaware of their underlying diabetic condition until diagnosed with DR.

**Figure 1:**
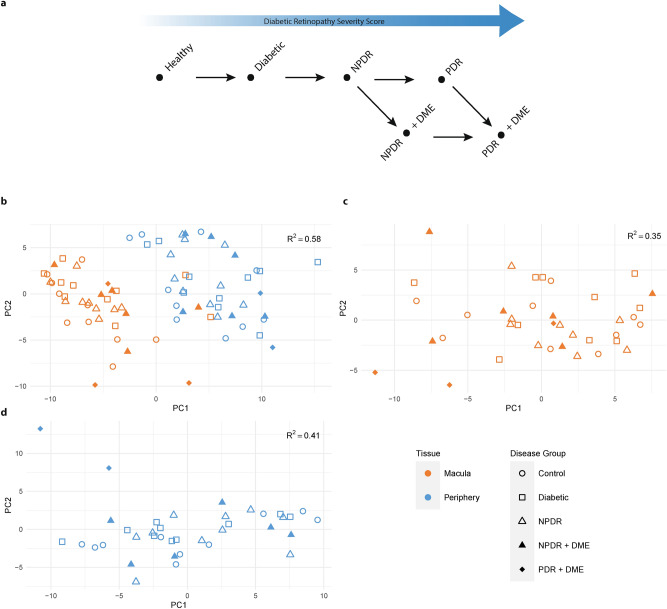
Gene expression differences between sample sites: (**a**) Schematic of disease progression in DR (NPDR: Non-Proliferative Diabetic Retinopathy, PDR: Proliferative Diabetic Retinopathy, DME: Diabetic Macular Edema). From left to right the Diabetic Retinopathy Severity Score (DRSS) increases. (**b**) Principal component analysis (PCA) scores of combined and processed mRNA and miRNA expression values for all samples. (**c**) PCA scores of macula samples only. (**d**) PCA scores of periphery samples only. In all cases, PCA was applied to the top 100 most variable features in each sample group (all, macula, periphery). Cumulative R^2^-values for two principal components are shown in the upper right corner.

The Diabetic Retinopathy Severity Score (DRSS)—based on data from the Early Treatment Diabetic Retinopathy Study^3^ (ETDRS)—allows for grading of DR severity, including differentiation between non-proliferative DR (NPDR) and proliferative DR (PDR). Diabetic macular edema (DME) may occur concomitantly to any stage of DR, and negatively affects a patient’s visual acuity (Fig. 1a).

Depending on disease severity, the current standard of care for DR is laser photocoagulation together with continued diabetes management and eye screening^4^. Recently, intra-vitreal injection of Vascular Endothelial Growth Factor (VEGF) neutralizing agents was established as a novel treatment for DR, DME, and other retinal diseases. An alternative treatment is provided by intravitreally administered corticosteroids. All current treatment options, however, exhibit significant shortcomings including unsatisfactory efficacy^5^, and high treatment burden, which emphasizes the need to investigate novel targeting options and provide alternative treatment avenues. In turn, a deeper, molecular understanding of the pathophysiological onset and progression of DR is necessary to foster the development of alternative therapeutic strategies.

Several transcriptomic studies analyzing retinae of animal models in the context of DR have been carried out, including Streptozotocin (STZ) induced diabetic rodents models^6–8^, and models of oxygen-induced retinopathy (OIR)^9,10^. Another focus has been the measurement of small non-coding RNA in blood of DR patients^11–15^. With the advent of single-cell RNA-Sequencing (RNA-Seq) technology, retinal gene expression has now also been resolved on a cellular level^16–19^. However, no disease-specific dataset exists for retinae with DR. Ishikawa et al. provided a small bulk RNA-Seq dataset of fibrovascular membranes (FVM) of patients with PDR, which were comprehensively analyzed^20–22^. Due to the invasive nature of collecting retinal tissue, obtaining well-characterized, treatment naïve samples from patients remains challenging. While in the case of age-related macular degeneration, transcriptomic analysis of human retina has led to important progress in the molecular understanding of disease etiology^23,24^, comparable data for patients with DR does not exist. Hence, detailed insight into the molecular mechanisms relevant for DR in humans remains incomplete.

Here we performed RNA-Seq analysis on a large number of human post-mortem retinal samples from patients diagnosed with different stages of DR: NPDR only, NPDR and DME, or PDR and DME. To ensure observed gene expression changes were associated with disease and not specific treatments, retinal samples, together with extensive clinical records, were obtained from individuals that had not received anti-VEGF or laser photocoagulation therapy. We further included diabetics found to be free of DR on their last examination, while age-matched healthy subjects served as control group. In addition to differential gene expression analysis, we identified coordinated expression changes associated with disease progression using a multivariate statistical model. While simultaneous measurement of mRNA and miRNA enabled us to investigate disease relevant miRNA/mRNA interactions, the integration of human retinal single cell RNA expression data further resolved cell specific RNA expression changes.

## Results

### Gene expression differs between macula and retinal periphery

We obtained human retina samples from two distinct retinal sample sites (macula, periphery) within a maximum time window for sample collection and preparation of 6 h post-mortem (Supplementary Tables S1 and S2). Extensive clinical records, together with the ophthalmologist’s grading of patient eyes according to the simplified DRSS classification, served as the basis to assign donors to one of three main disease groups: Diabetics without diagnosis of DR, patients diagnosed with NPDR but without DME, patients diagnosed with NPDR or PDR and DME (Fig. 1a; Table 1). Retinae from age-matched healthy donors were included as control. Each of the four patient groups (Healthy control, Diabetic, NPDR without DME, NPDR/PDR + DME) included 10 samples from the two different sample sites, for a total of 80 retina-derived samples. For additional donor and sample level characteristics please refer to Supplementary Figure S1.

**Table 1:** Overview table of collected post-mortem human retina samples: the study contains four main sample groups (healthy control, diabetics without diagnosis of DR, NPDR, and PDR). In addition, donors are subdivided according to the occurrence of DME. Samples were taken from both retinal macula and retinal periphery. Throughout the manuscript NPDR/PDR + DME denotes the group of all donors with DME diagnosis.

Disease group	Macula		Periphery	
	DME		DME	
	No	Yes	No	Yes
PDR	0	3	0	2
NPDR	10	7	10	8
Diabetic	10	0	10	0
Healthy	10	0	10	0

Total RNA-Seq resulted in approximately 69 million average reads per sample, with 92% uniquely mapped reads (Supplementary Figure S2a). Sequencing of small RNA produced on average 24.7 million reads with a unique mapping rate of 55% (Supplementary Figure S2b). Four samples were removed across both sequencing datasets: One due to failure to pass primary sequencing metrics QC and three additional samples as they showed increased sample correlation (Pearson correlation > 0.98, Supplementary Figure S2c), and originated from the same donor but different eyes. After gene filtering, we assigned 15,073 expressed mRNA and 256 miRNA for further analysis (Supplementary Figure S2d,e). Principal component analysis (PCA) showed a pronounced technical confounder effect on the level of mRNA, which we removed using surrogate variable analysis (See Methods and Supplementary Figure S2f,g). In addition, we corrected both miRNA as well as mRNA for age-related expression biases.

PCA of the processed and combined miRNA and mRNA expression revealed a clear separation of samples between macula and periphery (Fig. 1b), indicating the largest difference in gene expression between these retinal regions irrespective of disease status. Repeating the PCA analysis for each sample site separately, PDR + DME samples in both macula and periphery appear more distinct compared to samples from the remaining disease groups (Fig. 1c,d). However, also sample site specific PCA showed no further separation of remaining disease groups in the first two principal components.

### Differential gene expression changes are limited to late DR stages

**Figure 2:**
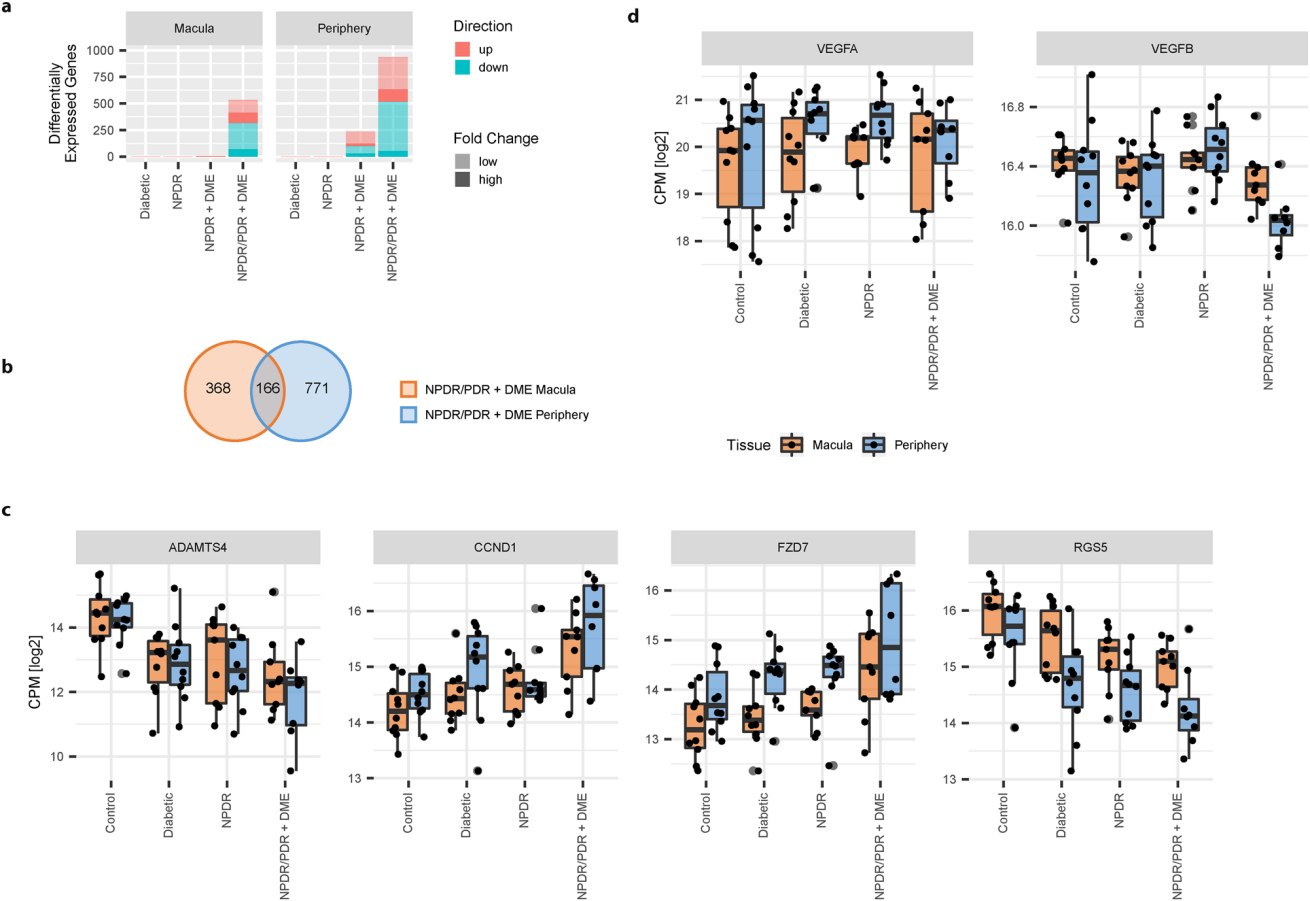
Significant changes in late stage disease progression: (**a**) Barplot with number of significant changes in RNA expression between disease groups and healthy control (BH adjusted *p* value < 0.05). Gene expression changes with fold-change below 50% are transparent. Up- and down-regulation are shown in red and blue respectively. (**b**) Venn diagram showing the overlap between macula and periphery of differentially expressed transcripts identified in the NPDR/PDR + DME group. (**c**) Log2 CPM expression values (black dots) of significantly changing genes identified from NPDR/PDR + DME samples with strongest mean fold-change between macula and periphery. Black line denotes median log2 CPM. Boxplot lower and upper hinges correspond to the first and third quartiles respectively. Whiskers extend to the largest expression value no further than 1.5 interquartile range from the hinge. Outliers are shown in transparent gray. (**d**) Log2 CPM values (black dots) of VEGFA and VEGFB expression throughout subsequent disease groups. Boxplot specifications correspond to those given in the previous plot.

We checked for significant expression changes (BH adjusted *p* value < 0.05) between the different disease groups and healthy control. While our analyses did not identify significant retinal expression changes in either age-matched diabetics without DR or patients with NPDR but without DME (Fig. 2a, Supplementary Figure S3a), we observed a number of significant expression changes in peripheral retinal samples from NPDR patients diagnosed with DME (NPDR + DME). Grouping NPDR and PDR samples with occurrence of DME (NPDR/PDR + DME), we detected 534 and 937 differentially expressed (DE) transcripts in macula and periphery samples respectively. However, the magnitude of these expression changes was low, with relative changes in many genes below 50%.

Comparing significant expression changes in the NPDR/PDR + DME groups, we found them to be mostly sample site specific: Out of all 1305 identified transcripts (1269 mRNA and 36 miRNA), only 166 overlapped between both sample sites (Fig. 2b). On the other hand, inspecting relative expression changes among all transcripts, we could observe that fold changes in the NPDR/PDR + DME group aligned between macula and retinal periphery (Supplementary Figure S3b,c). Accordingly, we did not identify any inverse changes in gene expression, i.e. RNA showing different directions of expression change between the two sample sites in any of the defined disease groups (Pearson correlation coefficients macula vs periphery: 0.58–0.65). Inspecting expression patterns of differentially expressed transcripts throughout subsequent disease progression stages, we further observed that many significant changes in late disease stages were accompanied by non-significant changes in previous disease stages (Supplementary Figure S3c,d).

Among the genes with strongest significant expression changes, we found ADAMTS4 (ADAM Metallopeptidase With Thrombospondin Type 1 Motif 4), CCND1 (Cyclin D1), FZD7 (Frizzled Class Receptor 7), and RGS5 (Regulator Of G Protein Signaling 5) (Fig. 2c). Although increase of VEGFA (Vascular Endothelial Growth Factor A) expression is considered central to DR, neither VEGFA nor VEGFB appeared to be upregulated in disease progression (Fig. 2d).

### Multivariate modeling identifies disease progression associated expression changes

The lack of significant RNA expression changes in early disease stages relative to healthy samples motivated us to search for progressive changes throughout consecutive disease stages. With this aim in mind, we applied sparse partial least squares (SPLS) regression models^25^ for each macula and periphery samples to identify mRNA and miRNA that are predictive for the subsequent disease stages (Healthy, Diabetic, NPDR, NPDR + DME; Supplementary Figure S4a). We further permitted additional flexibility in the regression models, in the sense that we did not specify the relative order or distance between disease groups, but only start- (Healthy control) and end-point (NPDR + DME) of disease progression.

**Figure 3:**
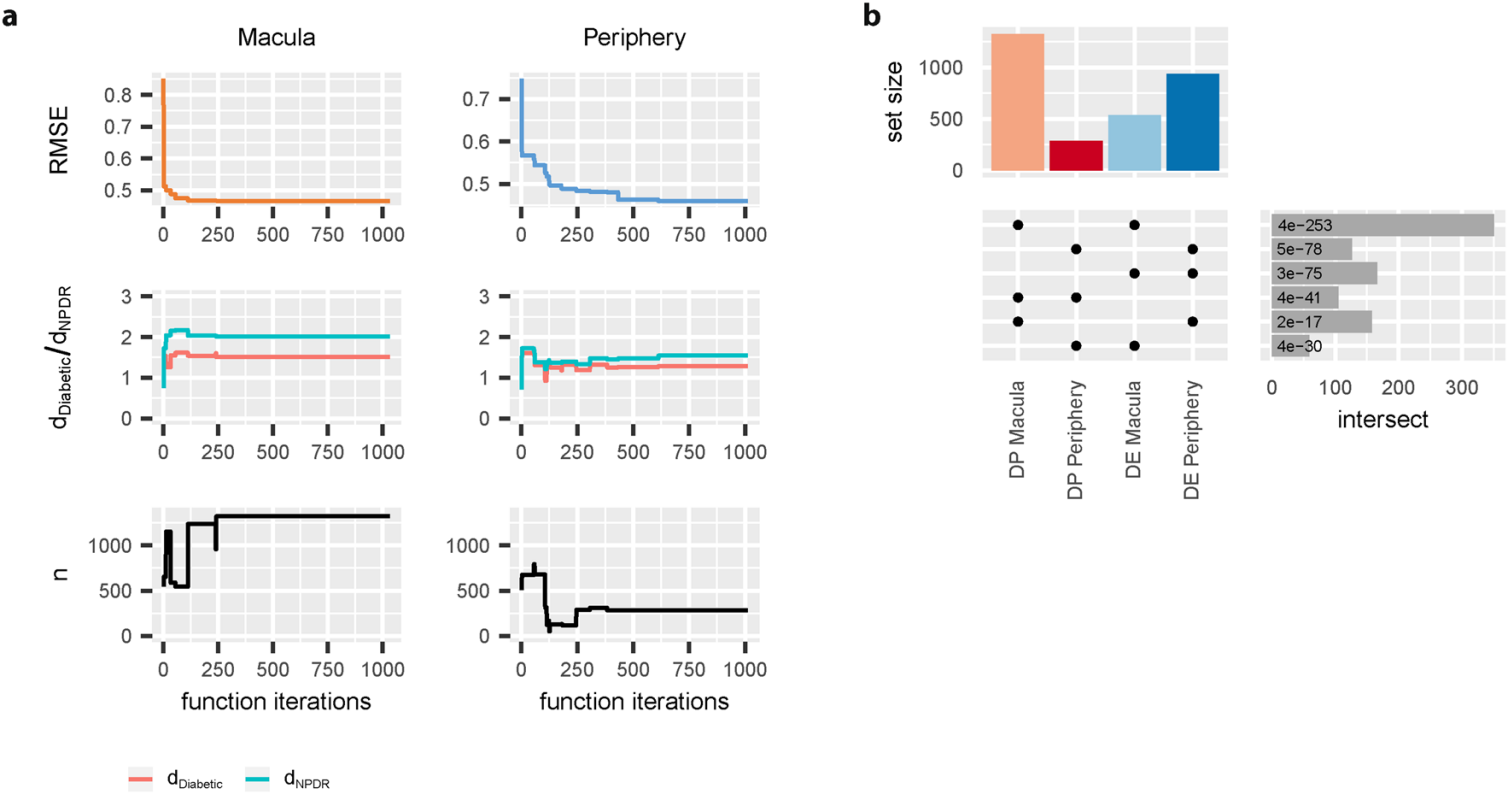
Identification of disease progression genes: (**a**) Convergence properties of sparse partial least squares regression model to identify disease progression (DP) transcripts: Minimum Root Mean Squared Prediction Error (RMSE) of the regression model after each function iteration for macula and periphery (upper panel). Value of d_Diabetic_ and d_NPDR_ model hyperparameters for the best model fit at each function iteration (middle panel) for macula and periphery samples. Number of selected transcripts (n) for the best model fit at each function iteration (lower panel). (**b**) Comparison of identified DP RNA with RNA identified from differential gene expression analysis (upper panel) and corresponding two-set intersects (lower right panel). Values in the lower right panel denote BH adjusted *p* values (hypergeometric test).

As a result of fitting the model to the expression data, we accurately captured the assumption that the NPDR samples are more severe in terms of disease pathology compared to diabetic samples (Fig. 3a—middle panel). The fitted models further identified 1322 macula and 285 periphery transcripts displaying progressive changes according to consecutive disease stages (Fig. 3a—lower panel), herein referred to as “disease progression” (DP) mRNA or miRNA. Combining DP transcripts from both sample sites (union), we obtained 1502 genes, indicating that 105 appeared in both macula and periphery (intersect, Fig. 3b). Although the overlap between transcripts identified from both sample sites was significant (4.27 fold-enrichment, hypergeometric test, BH adjusted *p* value = 3.7e−41), at the same time a substantial fraction of DP transcripts was identified by one sample site only. This suggests once again that, while some commonalities exist, the cellular responses of the peripheral and macular retina to diabetic stress appear to be different to some extent. We further observed a significant overlap between identified DP and DE RNA (intersect: 351 macula, 126 periphery), likely due to the correlation between disease progression and occurrence of DME. Similar to significant expression changes in the NPDR/PDR + DME groups, we did not observe inverse changes in expression of DP transcripts between macula and periphery (Supplementary Figure S4b).

### DE and DP transcripts reveal separate sets of disease relevant pathways

**Figure 4:**
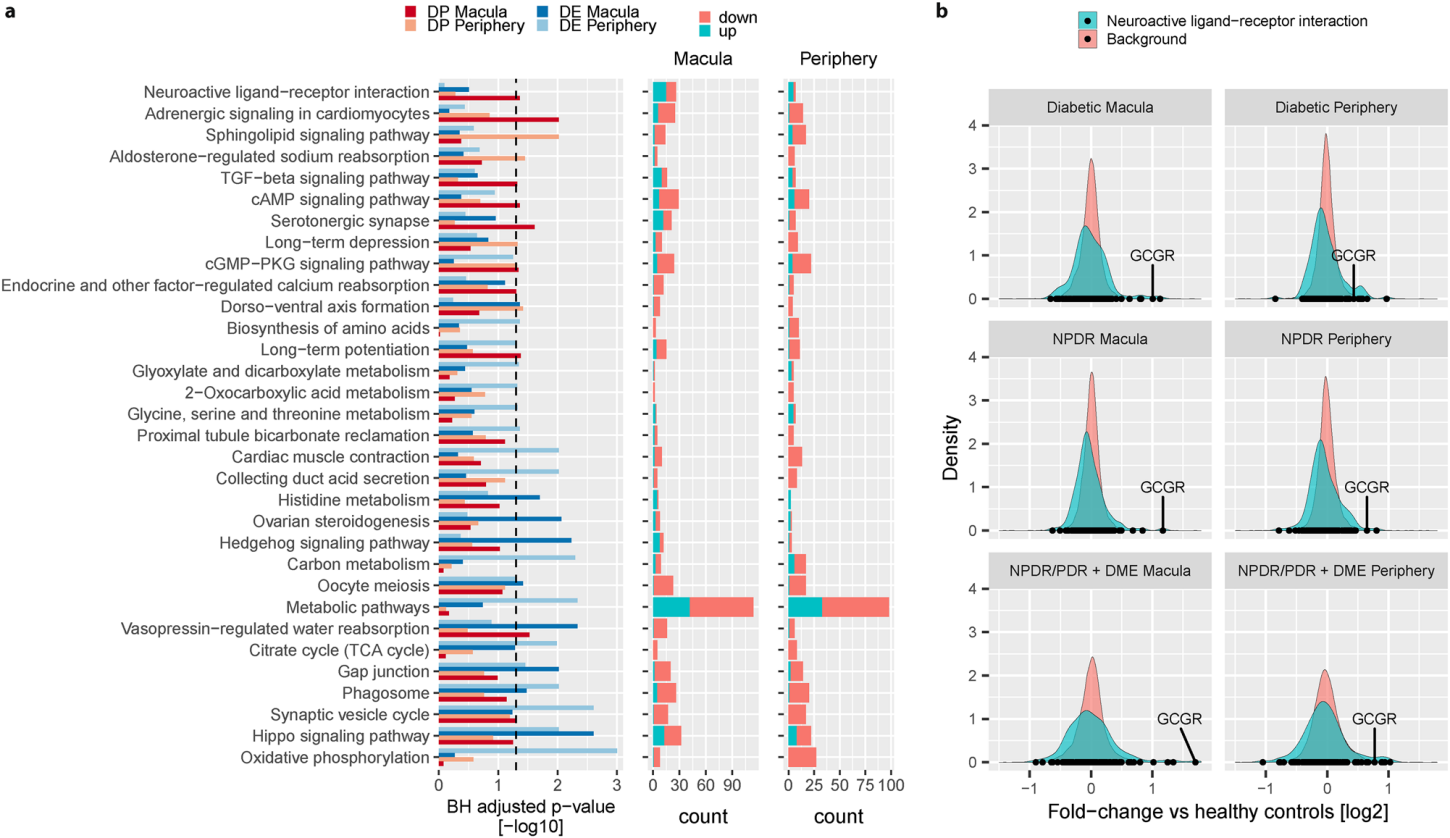
Molecular pathways associated with different disease stages of DR: (**a**) Barplot showing BH-adjusted *p* values of KEGG 2016 pathway enrichment analysis for each the four identified groups of disease-associated transcripts (left panel, Oxidative phosphorylation BH adjusted p value = 1.15e−07). Only pathways with BH-adjusted *p* value < 0.05 are shown. Middle and right panel indicate the direction of change in NPDR/PDR + DME samples vs healthy control for each of the significantly changing transcripts included in a given pathway (Middle panel: Macula; Right panel: Periphery). (**b**) Distribution of fold-changes for each sample group vs healthy controls for transcripts associated with the Neuroactive ligand–receptor interaction pathway. Black dots indicate fold-changes of individual genes in the Neuroactive ligand–receptor interaction pathway. As example, we specifically plot Glucagon Receptor (GCGR). The background distribution shows fold-changes [log2] of all transcripts not included in the pathway.

To gain insight into the molecular mechanisms involved in different stages of DR, we checked for pathways enriched for disease-associated mRNA and miRNA. From this analysis we identified 31 pathways significantly associated (hypergeometric test, BH adjusted *p* value < 0.05) with at least one of the four sets of identified transcripts, either differentially expressed in NPDR/PDR + DME samples or identified as disease progression associated (Fig. 4a). Although significant changes in the NPDR/PDR + DME and disease progression groups partially overlapped, pathways associated with each group were not identical. Rather, each of the identified sets showed a distinct overlap with different pathways.

For instance, we found a significant enrichment between peripheral DP factors and the sphingolipid signaling pathway. The enrichment analysis further showed a significant overlap between TGF-β signaling and DP transcripts identified from macula samples. Interestingly, cGMP-PKG signaling was significantly enriched for DP RNA of both macula and periphery, suggesting a role for this pathway in both retinal regions. Vasopressin-regulated water reabsorption seemed to be particularly important for the retinal macula, as it showed significant overlap to both DP and DE transcripts identified from these samples.

Three pathways showed a significant enrichment for DE miRNA and mRNA for both macula and periphery: Phagosome, gap junction, and hippo signaling pathway. This observation points to relevance of these pathways particularly in late disease stages. The pathway observed with strongest overall enrichment was oxidative phosphorylation, which was significantly enriched for DE genes in the periphery (24 genes, 4.2 fold-enrichment, hypergeometric test, BH adjusted *p* value = 1.15e−07).

To elucidate the effect of disease on the identified pathways, we further investigated the direction of expression changes for each affected pathway (Fig. 4a—middle and right panel). The hippo signaling pathway included both significantly up- and down-regulated transcripts. Similarly, gap junction signaling exhibited regulation in both directions: Components of the tubulin complex were consistently downregulated, while for example the genes PDGFB (Platelet-derived growth factor subunit B) and ADCY6 (Adenylyl cyclase type 6) were observed to be upregulated.

As mentioned above, we did not detect any significantly differentially expressed transcripts in early disease stages. In order to identify pathways relevant for early disease progression, we therefore tested which pathways displayed a significant difference in their fold-change distribution compared to background (GAGE analysis, BH corrected *p* value < 0.05; Table 2). Remarkably, we found specific pathways consistently affected throughout early as well as late disease stages. The neuroactive ligand–receptor interaction pathway showed significant difference in fold-change distribution in the group of diabetic patients, but also in NDPR as well as NPDR/PDR + DME groups (Fig. 4b). In particular, GCGR (Glucagon Receptor) displayed high positive fold-changes persisting throughout the different disease stages. On the other hand, complement and coagulation cascades showed a significant alteration of its fold-change distribution only during late stages of DR (NPDR/PDR + DME samples) potentially suggesting an important role for fibrosis in late DR.

**Table 2:** Fold-change based pathway enrichment analysis: table shows results of the fold-change based pathway enrichment analysis conducted by GAGE. Group refers to the sample group for which fold-changes compared to healthy control samples were calculated. Set Size denotes the number of transcripts common in the 15,371 expressed transcripts in this study and the indicated KEGG set.

Pathway	Group	Tissue	Set size	BH adjusted *p* value
cAMP signaling pathway	NPDR/PDR + DME	Macula	145	0.015
Complement and coagulation cascades	NPDR/PDR + DME	Macula	32	0.000
Complement and coagulation cascades	NPDR/PDR + DME	Periphery	32	0.018
Cytokine–cytokine receptor interaction	Diabetic	Periphery	83	0.017
Cytokine–cytokine receptor interaction	NPDR/PDR + DME	Macula	83	0.035
ECM–receptor interaction	Diabetic	Macula	58	0.008
Hematopoietic cell lineage	Diabetic	Macula	24	0.021
Neuroactive ligand–receptor interaction	Diabetic	Macula	120	0.008
Neuroactive ligand–receptor interaction	Diabetic	Periphery	120	0.001
Neuroactive ligand–receptor interaction	NPDR	Macula	120	0.042
Neuroactive ligand–receptor interaction	NPDR	Periphery	120	0.001
Neuroactive ligand–receptor interaction	NPDR/PDR + DME	Macula	120	0.005
Protein digestion and absorption	NPDR/PDR + DME	Macula	47	0.010

### Combined analysis of miRNA and mRNA expression data highlights the role of miRNA in DR

**Figure 5:**
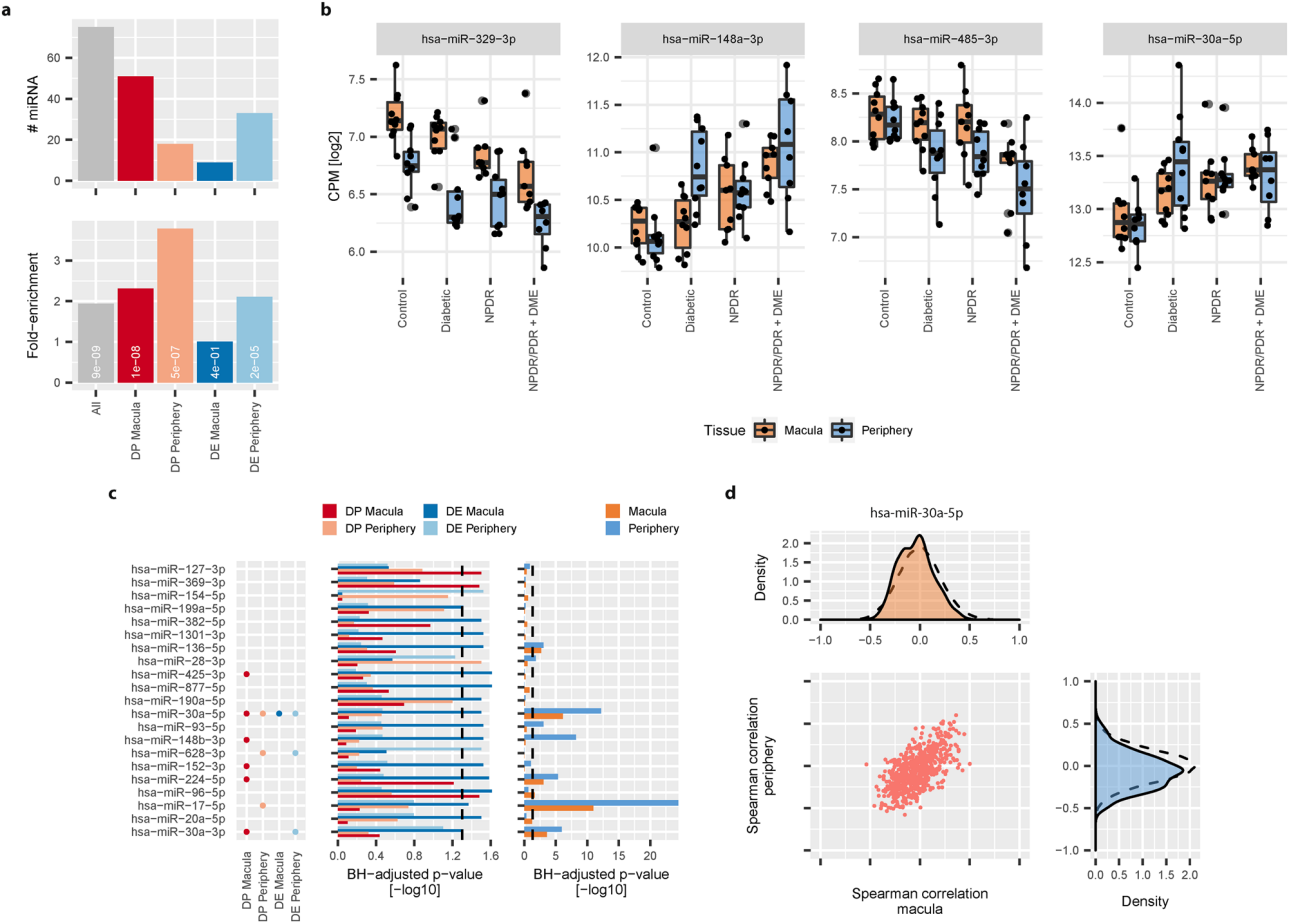
Integrated analysis of miRNA and mRNA expression: (**a**) Number of miRNA in all identified disease-associated transcripts (upper panel, gray bar) or in each group of identified disease-associated transcripts. Lower panel shows relative enrichment (observed vs expected) of miRNA in each of the defined disease-associated groups. White labels correspond to BH-corrected *p* values of a hypergeometric test. (**b**) Log2 CPM expression of miRNA identified in all four groups of disease-associated transcripts (black dots). Black line denotes median log2 CPM. Boxplot lower and upper hinges correspond to the first and third quartiles respectively. Whiskers extend to the largest expression value no further than 1.5 interquartile range from the hinge. Outliers are shown in transparent gray. (**c**) Barplot of BH-adjusted *p* values [− log10] of miRNA target enrichment analysis for each of the four identified groups of disease-associated genes (left panel). Only miRNA with significant enrichment of their target genes in any of the four defined groups of disease-associated mRNA are shown. Dashed line indicates the level of significance chosen (BH-adjusted *p* value < 0.05). Right panel shows BH-adjusted *p* values [− log10] of a Kolmogorov–Smirnov test for negative skew in correlation between miRNA and target mRNA expression (Orange: Macula samples, Blue: Periphery samples). (**d**) Distribution of Spearman correlation values between miR-30a-5p and its putative target genes in either macula (upper panel) or periphery samples (right panel). Dashed line corresponds to background distribution of 100.000 correlations between non-associated miRNA/mRNA pairs. Middle panel represents the scatter plot for individual Spearman correlation values between miR-30a-5p and its targets.

The measurement of both miRNA and mRNA in human retinal samples provided us with the unique opportunity to investigate potential disease relevant miRNA/mRNA interactions. Within all identified disease-associated transcripts (union of DE and DP transcripts from both sample sites, 2315 transcripts), we found 75 miRNA (Fig. 5a). The relative fraction of miRNA in identified disease-associated transcripts was largest in the set of DP RNA identified from periphery samples. Four out of 75 identified disease-associated miRNA showed progressive expression changes as well as differential expression in the NPDR/PDR + DME group in both macula and periphery samples (Fig. 5b).

Apart from significant or progressive expression changes, an enrichment of miRNA target genes within the sets of disease-associated mRNA may further strengthen the evidence for disease relevance of miRNA. Accordingly, we were able to identify 21 miRNA out of all expressed miRNA with significant enrichment (hypergeometric test, BH adjusted *p* value < 0.05) of their putative target genes among the different sets of identified disease-associated genes (Fig. 5c—middle panel). miRNA miR-96-5p showed enrichment of its target genes in both DE as well as DP genes of the macula, while for target genes of all other identified miRNA we only observed enrichment in one group of disease-associated genes. Interestingly, eight out of the 21 miRNA with significant enrichment of target genes were themselves either differentially expressed in late DR or showed progressive change in one of the sample sites (Fig. 5c—left panel). In periphery samples, miR-628-3p showed progressive expression changes as well as differential gene expression in the NPDR/PDR + DME group. In addition, target genes of the same miRNA showed a significant overlap with differentially expressed genes of the same sample site. In contrast, miR-30a-5p was identified in all four defined sets of disease-associated genes, while its targets only showed significant enrichment in differentially expressed genes of the macula.

Due to the inhibitory relationship of miRNA–mRNA interactions, often a negative correlation between miRNA and their targets is assumed. We therefore checked for a negative skew in correlation values between miRNA and their putative targets (Supplementary Figure S5a). Accordingly, we identified 10 miRNA with a significant negative shift (Kolmogorov–Smirnov-Test, one-sided, BH adjusted *p* value < 0.05) in the distribution of correlation values in either macula or periphery (Fig. 5c—right panel, Fig. 5d), which also showed an enrichment of their putative targets among the groups of disease-associated genes.

Taken together, five miRNA (miR-148b-3p, miR17-5p, miR224-5p, miR30a-3p, and miR30a-5p) were identified as disease-associated miRNA, displayed enrichment of their putative targets in disease-associated genes, and showed a significant negative shift in correlation with their target genes.

### Integration of retinal single cell RNA-Seq data provides evidence for loss of RGCs in early DR

**Figure 6:**
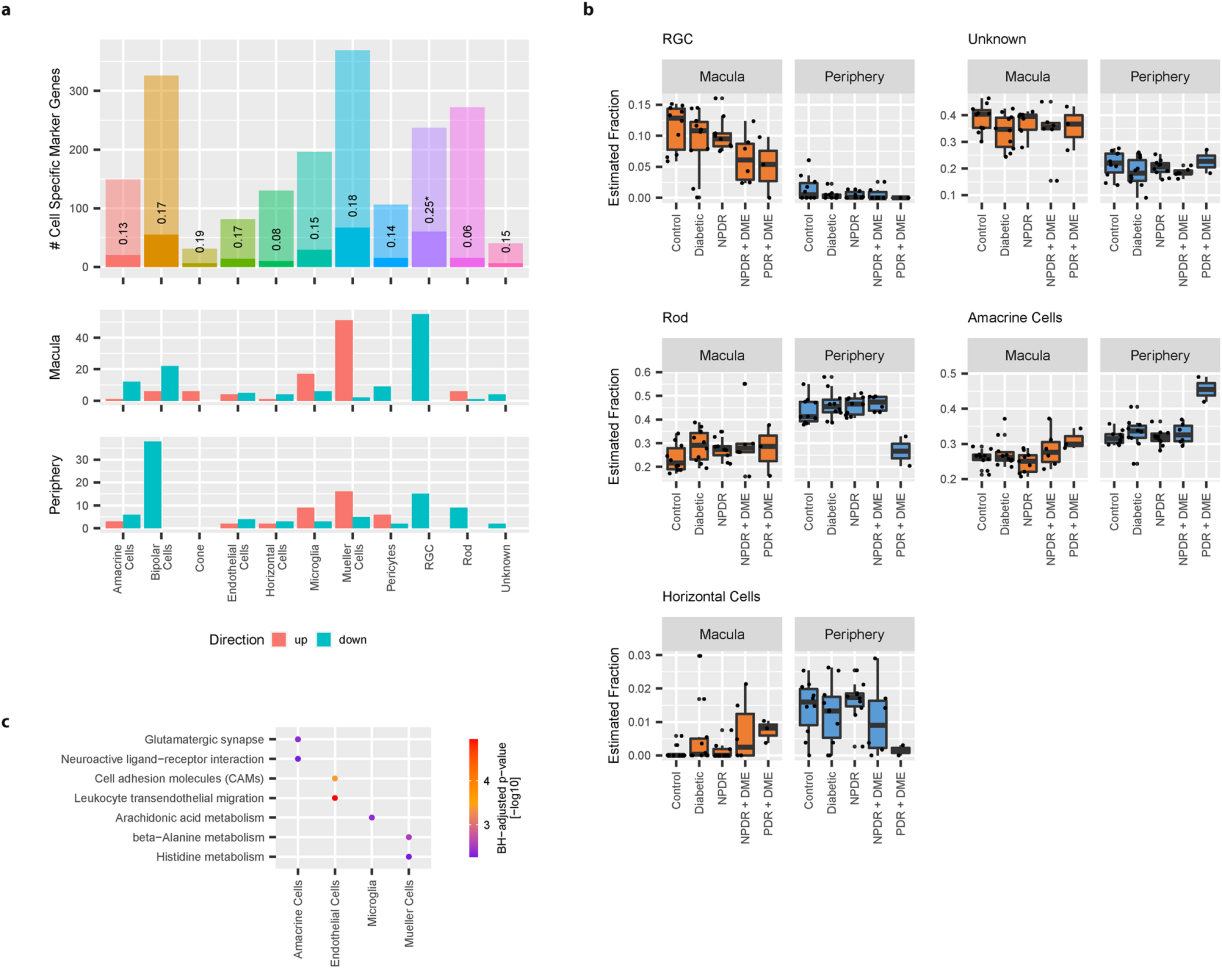
Cell specific expression of disease-associated RNA: (**a**) Enrichment of disease-associated genes with cell type specific genes identified from single cell RNASeq data. Barplots indicate the number of cell type specific marker genes identified for each cell type (upper panel). Non-transparent area of bars show the overlap of disease-associated genes (union of DE and DP genes from macula and periphery) with cell type specific marker genes. Middle and lower panel correspond to the direction of expression changes in NPDR/PDR + DME samples vs healthy controls of disease-associated genes (Middle panel: Macula; Lower panel: Periphery). (**b**) Estimation of cell type abundances from bulk retinal samples using deconvolution. Black dots show predicted fraction of different cell types in each of the bulk retinal samples. Black line indicates the median fraction of different cell types in each disease group. Boxplot lower and upper hinges correspond to the first and third quartiles respectively. Whiskers extend to the largest cell type fraction no further than 1.5 interquartile range from the hinge. Outliers are shown in transparent gray. (**c**) Enrichment of cell type specific disease-associated genes with molecular pathways. Plot shows significant (Hypergeometric test, BH-adjusted *p* value < 0.01) associations between cell type specific disease-associated genes and KEGG 2016 pathways. Color indicates BH-adjusted *p* values [− log10].

Although informative, the analysis of expression changes from bulk tissue samples on its own does not resolve cell type associated changes in the progression of DR. However, recently a number of single cell RNA-Seq (scRNA-Seq) studies of the human retina have been published^16–19^, providing the opportunity to investigate cell specific expression of disease related genes. Comparing cell specific marker genes for the different cell types identified from the Voigt et al. 2019 retina scRNA-Seq data (Supplementary Figure S6a), we observed that disease related genes (DE and DP from both sample sites) were significantly overrepresented in marker genes related to Retinal Ganglion Cells (RGCs) (hypergeometric test, BH-adjusted *p* value: 1.03e−4; Fig. 6a—upper panel). Virtually all of these 60 genes displayed a decrease in expression levels in late disease stages (Fig. 6a—middle and lower panel, Supplementary Figure S6b), hinting at a general loss of RGCs during disease progression.

Investigating this further, we estimated the relative fractions of each retinal cell type present in bulk RNA-Seq samples using the deconvolution method MuSiC^26^. In accordance with the general decrease in RGC marker genes, the deconvolution approach predicted a continuous decrease in the relative abundance of the RGCs throughout the different stages of disease progression (Fig. 6c).

While downregulation of most RGC specific marker genes could be attributed to a loss of RGCs in disease progression, marker genes specific to the other retinal cell types showed both up- and down-regulation of disease-associated genes in NPDR/PDR + DME samples. For example, although a loss of Müller cells in diabetic retinopathy has been proposed^27^, the observation of positive gene expression changes in genes specific to this cell type suggests that a general loss of this cell type is less likely. We followed up on the role of cell type specific disease-associated genes in the different cell types again using pathway enrichment analysis (Fig. 6c). Specifically in Müller cells, our results pointed to a functional role of this cell type in processes related to histidine and β-alanin metabolism. Endothelial cells are central to disease pathology of DR, as they mediate vascular function and regulate the retinal environment. Disease-associated genes specific to this cell type showed a significant enrichment with genes related to cell adhesion molecules and leukocyte transendothelial migration.

## Discussion

RNA sequencing presents a valuable method to gain a detailed and unbiased understanding of the transcriptomic basis of human disease. However, high quality samples of human origin are difficult to obtain. While Ishikawa et al. report the microarray analysis of six FVMs^20^, the magnitude of this dataset remains limited. In addition, FVMs display pathologic vascular and fibrotic tissue, only present in patients with PDR. Additional currently available transcriptomic studies in the context of DR are restricted to animal models, such as STZ rat or OIR mouse. To fill this gap in the human data landscape of retinal pathologies, we collected an extensive and unique RNA-Seq dataset of post-mortem retinal samples from patients with DR.

In addition to the limited availability of suitable human retinal samples, observed heterogeneity in disease etiology^28^, as well as genetic and environmental variation in the donor population, further complicate the analysis of transcriptome studies. Using differential expression analysis, we were able to meaningfully characterize disease mechanisms in particular associated with late stage DR. To simultaneously access information from all collected retinal samples, we complemented this analysis by applying a multivariate regression model. In contrast to differential gene expression analysis, such a model can capture coherently changing sets of genes with potentially low-fold changes, instead of isolated changes in individual disease groups. Interestingly, although our model formulation did not impose a fixed sequence of disease stages, the model correctly predicted their relative order based only on the available gene expression data. We hypothesize that genes identified by the regression model contain causal mechanisms contributing to disease progression and may provide useful biomarkers for its onset.

Although molecular studies and current treatments have shown that VEGFA is central to the etiology of DR^29–32^, VEGFA was neither differentially expressed nor did it show progressive expression changes in our analysis. One explanation for this observation could be restricted VEGFA upregulation to local (capillary) focal points or cell-types with limited retinal abundance. While also post-transcriptional regulation of VEGFA may be considered, additional mechanisms or pro-angiogenic factors other than VEGFA are likely to be involved in the onset and progression of DR.

Here PDGFB, the inhibition of which is considered a potential therapeutic approach for ocular neovascularization, displayed significant upregulation in late stage DR, and multiple clinical trials investigating the effect of PDGF antagonists have been pursued^33,34^. PGF (Placental Growth Factor), a known VEGF homologue and secondary target of anti-VEGF treatment, did not show significant change in any of the disease groups, but instead displayed progressive changes throughout the different disease stages in macula samples, highlighting the value of the multivariate regression model to identify subtle but important gene expression changes.

In addition to alternative vascular mechanisms, post-transcriptional regulation of VEGF activity may be important. For instance, anti-angiogenic properties have been reported for ADAMTS4 through the inhibition of VEGF dependent VEGFR (VEGF-Receptor) phosphorylation in human dermal microvascular endothelial cells^35^, suggesting a potentially increased responsiveness to VEGF under conditions of low ADAMTS4 expression. In our data, ADAMTS4 showed progressive expression changes in addition to significant down-regulation in both sample sites, and presented one of the strongest changes in diabetic samples.

While previously mainly considered a vascular disease, attention in DR has recently shifted to additional mechanisms and cell-types. In alignment with this new understanding, we identified a number of genes associated with potential alternative pathological processes. Prominent early changes were for example noted for CRYAA2 (Crystallin Alpha A2) and GCGR, both involved in metabolic processes. CRYAA2 has been described as a retinal response mechanism against environmental and metabolic stress, and is potentially involved in protection against loss of RGCs^36^, while GCGR is known for its connection to various diabetic complications^37^.

Moving away from singular expression changes, enrichment analysis suggested a number of functional pathways involved in DR. Three pathways showed significant overlap with differentially expressed genes in late DR in both macula and periphery: Phagosome, gap junction, and hippo signaling pathway. Notably, all three pathways have previously been discussed in context of DR^38–42^. On the other hand, we observed an enrichment of disease progression transcripts from both macula and periphery with cGMP-PKG signaling, which is known to be affected by retinal hypoxia^43^. Further examples of identified pathways previously studied in the context of DR include sonic hedgehog signaling^44^, sphingolipid signaling pathway^45,46^, and oxidative stress^47^, adding to the good agreement between our findings and those reported in the experimental literature.

Important for therapeutic intervention, but also to assess disease risk, are accessible biomarkers, such as miRNA present in the blood of diabetic or DR patients^48^. A study investigating circulating vesicles in DR pointed to a number of differentially expressed miRNA, including a significant decrease of miR-150-5p^14^. A progressive downregulation of miR-150-5p in both retinal regions with advancing disease stages was also visible in our data. Based on blood samples from type 1 diabetics diagnosed with NPDR, a recent study has linked miRNA miR-27b-3p and miR-320a to progression of DR^49^. Although miR-320a showed no significant expression change in the transcriptomic data, we confirmed disease progression associated expression of miR-27b-3p in the macula. Liang et al. identified 10 miRNA significantly different in blood of type 2 diabetic patients with DR compared to diabetic patients without DR, including miR-148a-3p and let-7a-5p^13^. They continued to show that overexpression of let-7a-5p significantly increased the proliferation of human retinal microvascular endothelial cells. Both miR-148a-3p, as well as let-7a-5p showed significant upregulation and progressive expression in patient retina analyzed here.

From a treatment perspective, miRNA present an interesting therapeutic intervention strategy as they typically influence the expression of multiple genes, are easy to synthesize, and show limited toxicity. For example, miR-329 was shown to exhibit anti-angiogenic properties by suppressing expression of CD146, an adhesion molecule that acts as a co-receptor for VEGFR2^50^. Consequently, treatment of mouse models of pathological angiogenesis with miR-329 significantly reduced retinal neovascularization. Accordingly, we detected a progressive down-regulation of miR-329 in subsequent disease stages also in human samples. While expression changes between studies of peripheral blood and retina match for specific miRNA, this was certainly not the case for all miRNA. In the future, longitudinal studies should assess the potential of identified miRNA to stratify diabetic patients with high-risk of developing DR.

By investigating cell-specificity of DR associated genes in retinal scRNA-Seq data from healthy donors, a recent study showed expression of known DR genes mainly present in RGCs^51^. Here we show that this observation is likely caused by continuous down-regulation of RGC-specific genes in the progression of DR, hinting at a general loss of this cell type. In our analysis, partial loss of RGCs was already visible in diabetic patients without diagnosis of DR. This agrees with optical coherence tomography studies, which displayed RGC damage already at this somewhat pre-pathological stage of DR^52^. In addition to a general reduction of entire cell types, we observed more gradual pathway and cell specific alterations in disease progression, namely Müller cell mediated changes in β-alanine and histidine signaling, and expression changes in cell adhesion molecules in endothelial cells.

Collection of high quality retinal samples from diseased patients remains a challenging task. While the human retinal transcriptome data and its analysis presented in this study confirm many known aspects of DR pathology, and provide novel insights into the molecular and cellular basis of DR, we believe additional patient samples and metadata could prove useful. In particular, retinal samples from donors with late stage DR may help to separate the overlapping effects of PDR and DME and add statistical power to the presented computational analysis. Prospective longitudinal collection of patient data, such as OCT images, and blood samples, may further improve DRSS grading and aid the identification of biomarkers reflecting disease outcome. While in the past, studies have focused on the impact of DR on the neural retina, large-scale transcriptomic studies on the retinal pigment epithelium are currently still missing. In the context of disease progression markers, but also in the light of the missing VEGF RNA-regulation observed in our study, measurement of additional large-scale data modalities such as proteomics and metabolomics should be considered. Here we believe that integrative machine learning efforts will be indispensable in order to manage the growing data complexity.

We believe that the presented transcriptome analysis of human retinae strengthens the current data landscape of vision-threatening eye diseases, serves to improve our understanding of the molecular basis of disease etiology, and offers potential strategies for future investigations into improved treatments of DR and DME. In the future, a detailed comparison between disease models and observed human expression changes in DR may help to evaluate the ability of animal models to reflect human disease, and hereby judge their utility in investigating therapeutic strategies in a pre-clinical setting.

## Methods

### Sample collection and RNA isolation

Post-mortem human eyes from 43 donors were obtained through the Iowa Lions Eye Bank (Coralville, IA) after written informed consent of the next-of-kin was obtained and preserved within 6 h post-mortem. Upon donor selection, we did not include donors with a known history of HIV, Hepatitis B or C, or those placed under hospital isolation precautions. Further, eyes from donors suffering from neurodegenerative diseases of unknown etiology were excluded. Donors were categorized into one of four disease groups: (a) diabetic donors with no apparent visual impairment or visible pathology of the retina at last eye exam, (b) donors diagnosed with non-proliferative diabetic retinopathy (NPDR) but without diabetic macular edema (DME), (c) patients diagnosed with NPDR with additional indication of DME, or (d) donors diagnosed with proliferative diabetic retinopathy (PDR) and DME. The diagnosis was based on the early treatment diabetic retinopathy study Diabetic Retinopathy Severity Score (ETDRS-DRSS). Additional retinal samples were taken from healthy donors to serve as the control group. The study was designed such that donors of all groups approximately matched in age and gender distribution. Note that none of the donors received treatment for their retinal indication. Potential independent treatments are listed in Supplementary Table S1. The study design was reviewed by the University of Iowa Institutional Review Board and found not to involve human subjects research.

From each donor group (healthy control, diabetic, NPPR, and NPDR/PDR + DME), 10 tissue samples were collected from the macula region as well as the retinal periphery. All tissue samples were taken with a 6 mm biopsy punch, snap frozen in liquid nitrogen, and preserved at − 80 °C until use. Both miRNA and total RNA was extracted using miRNeasy kits (Qiagen) after homogenization of the retina with Qiashredders (Qiagen). RNA was eluted into 30 μL of dH_2_O. RNA was quantitatively and qualitatively assessed using the fluorescence-based Broad Range Quant-iT RNA Assay Kit (ThermoFisher), and the Standard Sensitivity RNA Analysis DNF-471 Kit on a 48-channel Fragment Analyzer (Agilent), respectively. Concentrations averaged at 100 ng/µL while RIN ranged from 4 to 9.

### Transcriptome profiling with total RNA sequencing

Retina-derived RNA samples were normalized on the MicroLab STAR automated liquid platform (Hamilton). Total RNA input of 240 ng was used for library construction with the NEBNext Ultra II Directional RNA Library Prep Kit for Illumina #E7760, together with the NEBNext rRNA depletion Kit #E6310 upstream and the NEXNext Multiplex Oligos for Illumina #E7600 downstream (all New England Biolabs). The only deviation from the manufacturer’s protocol was the use of Ampure XP beads (Beckman Coulter) at the double-stranded cDNA purification step, instead of the recommended SPRIselect Beads. Final sequencing libraries were quantified by the fluorescence dye-based methodology High Sensitivity dsDNA Quanti-iT Assay Kit (ThermoFisher) on a Synergy HTX (BioTek). Total RNA libraries were also assessed for size distribution and adapter dimer presence by the High Sensitivity NGS Fragment DNF-474 Kit on a 48-channel Fragment Analyzer (Agilent). Sequencing libraries were normalized on the MicroLab STAR (Hamilton), pooled as two 40-plex pools and spiked in with PhiX Control v3 (Illumina). The two individual pools were then separately clustered on a cBot instrument with a HiSeq 3000/4000 PE Cluster Kit and subsequently sequenced on a HiSeq 4000 Sequencer (all Illumina) with dual index, paired-ends reads at 75 bp length (Read parameters: Rd1: 76, Rd2: 8, Rd3: 8, Rd4: 76) to reach a minimum depth of 50 million Pass-filter reads per sample.

### Library preparation and sequencing for small RNA

RNA sequencing libraries were prepared using QIASeq miRNA Library Kit (Qiagen) according to the manufacturer’s instructions. In brief, 200 ng of RNA was used for the 3′ Adapter Ligation to the RNA followed by the 5′ Adapter Ligation. The tagged RNA library was translated into cDNA using reverse-transcription primer that contain integrated UMIs (Unique Molecular Indices), a universal sequence was also added in reverse transcription (RT) that is recognized by sample indexing primers during library amplification. RT was followed by a cDNA cleanup. During PCR amplification specific index PCR reverse primer were used and RT product was enriched to the final cDNA library. This was followed by a size selection purification via Magnetic beads. The library concentrations were quantified with the Quant-iT PicoGreen dsDNA Assay Kit (Quant-iTTM) using CLARIOstar (BMG LABTECH) and the library quality was determined by checking cDNA fragment size using a High Sensitivity DNA Kit on the Agilent Bioanalyzer 2100 (Agilent). miRNA Libraries were normalized to 8 nM and subjected to cluster generation on a cBot system, followed by sequencing on an Illumina HiSeq4000 instrument (Illumina).

### Processing of mRNA reads

Due to insufficient quality according to primary RNA-Seq quality control metrics, sample 45 (NPDR, macula) was removed for subsequent analysis. Sequencing reads from the RNA-seq experiment were processed with a pipeline built upon the implementation of the ENCODE’ “Long RNA-seq” pipeline: Filtered reads were mapped against the Homo sapiens (human) genome hg38/GRCh38 (primary assembly, excluding alternate contigs) using the STAR aligner software^53^ (STAR version 2.5.2b) allowing for soft clipping of adapter sequences. For quantification of transcript levels the annotation files from Ensembl version 86 were used, which corresponds to GENCODE 25. Samples were quantified with the above annotations, using RSEM^54^ (RSEM version 1.3.0) and featureCount^55^ (featureCount version 1.5.1). Quality controls were implemented using FastQC^56^ (FastQC version 0.11.5), picardmetrics (picardmetrics version 0.2.4), and dupRadar^57^ (dupRadar version 1.0.0) at the respective steps.

Normalized log2 Counts Per Million (CPM) mRNA expression values were calculated using the voom function provided by the limma R package^58^ (limma version 3.44.3). In total seven sample pairs qualified as technical replicates as they were collected from the same donor and sample site, but different eyes (see Table S1 for details). We removed three of these samples (samples 65, 75, 79), which showed high correlation between log2 expression values. mRNA were filtered by minimum read counts using the filterByExpr function of the edgeR package^59^ (edgeR version 3.30.3) and. In addition, a Gaussian mixture model (mclust^60^ version 5.4.6) was applied, fitting two Gaussian distributions to mean CPM expression data. mRNA assigned to the cluster with higher mean were retained. The pcaMethods package^61^ (pcaMethods version 1.80.0) was used to calculate principal components of the mRNA (all features included), which we correlated with technical sample metrics derived from Star, fastQC, picardmetrics, or featureCounts. Surrogate variables describing the observed technical confounder effect were estimated using the sva R package^62^ (sva version 3.36.0). Finally, mRNA expression was corrected for donor age as well as the estimated surrogate variable. In more detail, we designed linear models with mRNA expression of each individual transcript as response, and donor age as well as the estimated surrogate variable as covariates (lm function, stats R package version 4.0.2). For each linear model, a t-test was performed on the covariate to assess its statistical significance. In case the p-value for a transcript was below 0.05, the expression values of that transcript were replaced by the residuals of the linear model plus the intercept of the model.

### Processing of miRNA reads

Sequenced read quality was checked with FastQC (FastQC version 0.11.5). Subsequently miRNA-sequencing read adapters were detected using minion and trimmed using reaper from the kraken package^63^ (kraken version 13–274). The trimmed reads were aligned using the STAR Aligner (version 2.3.0e) on the miRBase 21 reference. In the following miRNA read counts were quantified using subread^64^ (version 1.4.5-PR1).

As recent studies suggest active miRNA to require high expression levels^65^, the threshold for filtering expressed/active miRNA was set at voom normalized log2 CPM > 100 for any of the available samples. miRNA expression values were corrected for age using the same confounder correction method as described above. Identifiers of mature miRNA were mapped to their precursor using the miRBaseConverter package^66^ (miRBaseConverter version 1.12.0).

### Identification of disease-associated genes

Quantified expression data (counts) and normalized expression values have been uploaded to the GEO database (Accession number: GSE160310). Principal Component Analysis (PCA) of the merged miRNA and mRNA data was again carried out using the pcaMethods R package. Top 100 variable features (mRNA/miRNA) were used for dimensionality reduction. We used the lmFit function of the limma package to compare gene expression of defined disease groups (Diabetic, NPDR, NPDR + DME, PDR/NPDR + DME) to healthy controls and identify significantly changing transcripts (Benjamini–Hochberg (BH) corrected *p* value < 0.05). Log2 CPM expression values of differentially expressed transcripts in the NPDR/PDR + DME group were clustered using the degPatterns function made available by the DEGreport package^67^ (DEGreport version 1.24.1).

We identified disease progression associated RNA using a modified Sparse Partial Least Squares (SPLS) regression model available in the spls R package^25^ (spls version 2.2.3, Supplementary Figure S4a). As before, we analyzed macula and periphery samples separately. In order to fit the regression model, we converted disease group labels into numeric values: The numeric value of healthy control samples was set to 0, while that of NPDR + DME samples was set to 3. As the number of PDR samples was too low (Macula: 3; Periphery: 2), these samples were not included in the regression analysis. Numeric values for the Diabetic group (d_Diabetic_) and NPDR group (d_NPDR_) were not set a priori, but included as additional hyperparameters of the model. Ranges for d_Diabetic_ and d_NPDR_ were set to 0.1 and 2.9. We further ranked features (miRNA/mRNA) according to their association with the numeric target vector using spearman correlation. The number of top ranked features included in the model (n) was not fixed, but added as an additional hyperparameter of the model. The minimum number of features was set to 50, while the maximum number of features was 15,329. Native hyperparameters of the SPLS model include κ (concavity of the objective function), η (thresholding parameter), and K (number of hidden components). While κ was set to a fixed value of 0.5, the ranges for η and K were set to {0.1, 0.9} and {1, 12} respectively. All model parameters were estimated by minimizing the root mean squared prediction error resulting from a 5 × 20 cross-validation implemented using the caret R package^68^ (caret version 6.0.84). Minimization was carried out using scatter search optimization implemented in the MEIGOR R package^69^ (MEIGOR version 1.18.0). As the objective function is dependent on integer valued parameter values and evaluation of the cost function is computationally expensive, we did not include a local solver in the optimization procedure. The maximum number of function evaluations was set to 1,000, while additional parameters remained at their default values.

### Pathway enrichment analysis

KEGG pathway enrichment analysis for disease-associated transcripts was performed using a hypergeometric test. We chose all 15,329 expressed transcripts identified from the retinal samples to serve as background for the statistical test. KEGG 2016 gene sets with an overlap of less than 10 transcripts with the background set were removed from our analysis. Further, all pathways from pathway categories “Human Diseases”, and “Drug Development” were excluded. Multiple testing was corrected using the Benjamini–Hochberg procedure (stats R package version 4.0.2).Please note that KEGG pathway gene sets are potentially subject to license restrictions (https://www.kegg.jp/kegg/legal.html). The authors of this manuscript have purchased an end-user license.

Fold-change based pathway analysis was carried out using the gage R package^70^ (gage version 2.38.3) together with KEGG 2016 gene sets described above. Changes towards both directions were tested simultaneously (same.dir = FALSE). The compare option of gage was set to ‘unpaired’. Maximum set size was set to 2,000. All other algorithm options remained at their default values.

### Integrated mRNA/miRNA analysis

Enrichment of miRNA in disease-associated transcripts was assessed using a hypergeometric test. As background the set of all 15,329 expressed transcripts was chosen. Fold-enrichment refers to the ratio of observed vs expected number of miRNA. Multiple testing correction was applied using the Benjamini–Hochberg procedure.

Known interactions between miRNA and target genes were collected using the miRTarBase database^71^ queried on 29.07.2019 (miRTarBase release 7.0). Only interactions between expressed miRNA or mRNA were included. Enrichment between known targets of differentially expressed miRNA and disease-associated mRNA from each of the four identified groups was calculated using a hypergeometric test. As background the set of all 15,071 expressed mRNA was chosen. miRNA with less than 10 targets in the set of expressed mRNA were excluded from the analysis (249 miRNA included). Multiple testing correction was carried out using the Benjamini–Hochberg procedure.

Correlation analysis was performed using Spearman correlation (stats R package version 4.0.2). The distribution of correlation values between miRNA and their known targets was compared to the background distribution of correlations (100,000 samples) via a Kolmogorov–Smirnov test (stats R package version 4.0.2). miRNA with less than assigned 10 targets among expressed mRNA were excluded from this analysis (249 miRNA included). Multiple testing correction was performed using the Benjamini–Hochberg procedure.

### Integration of human retinal single cell RNA sequencing data

Normalized expression data of human retina single-cell expression published by Voigt et al.^16^ was downloaded from the Gene Expression Omnibus database under the accession number GSE130636. Cell specific marker genes were identified using the Seurat R package^72^ (Seurat version 3.2.2). More specifically, we made use of the FindMarkers function provided by the Seurat package, choosing ‘wilcox’ as the statistical method to identify genes showing significant increased expression. Only genes expressed in human retinal bulk data presented in this study were included in this analysis (13,484 genes). The threshold for cell specificity was set at a Bonferroni-corrected *p* value < 0.01 and only markers unique to a single cell type were considered. All other parameters of the method were set to their default values. In order to compare disease-associated genes (DE and DP gene from macula and periphery) with cell type specific marker genes we performed a hypergeometric test, corrected via the Benjamini–Hochberg procedure. As background, the intersect between expressed genes in the human retina bulk data presented in this study with genes present in the human retina single-cell expression data was chosen.

The deconvolution analysis was performed using the MuSiC R package^26^ (MuSiC version 0.1.1). As reference data we utilized normalized expression values provided by the human retina single-cell RNA sequencing data mentioned above. Single cell expression data was down-sampled to include a maximum number of 500 cells from each cell type (Amacrine cells: 98; Bipolar cells: 500; Cone: 304, Endothelial cells: 254, Horizontal cells: 82; Microglia: 154; Mueller cells: 500; Pericytes: 111; RGC: 326; Rod: 500; Unknown cell type: 494).

Enrichment analysis for cell type specific disease-associated genes with KEGG 2016 pathways was carried out using a hypergeometric test. Prior to this Ensembl gene IDs were mapped to HGNC gene symbols using Ensembl version 86. As background we chose all genes expressed in both bulk and single cell RNA expression data (13,464 genes). KEGG 2016 pathways with an overlap of less than 10 transcripts with the background set were removed from the analysis. Pathway categories “Human Diseases”, and “Drug Development” were excluded. Multiple testing correction was performed using the Benjamini–Hochberg procedure.

### Ethics declaration

Post-mortem human eyes were obtained through the Iowa Lions Eye Bank (Coralville, IA) after written informed consent of the next-of-kin was obtained. The study design was reviewed and approved by the University of Iowa Institutional Review Board. All methods were carried out in accordance with relevant guidelines and regulations.

## Supplementary Information

Supplementary Figures.

Supplementary Table 1.

Supplementary Table 2.

## Data Availability

Code associated with this manuscript has been uploaded to https://github.com/bi-compbio/diabetic_retinopathy_iowa.
