# Supplementary material for: In-depth transcriptomic analysis of human retina reveals molecular mechanisms underlying diabetic retinopathy
Source: Sci Rep. 2021 May 18;11:10494. doi: 10.1038/s41598-021-88698-3 (PMC8131353; doi:10.1038/s41598-021-88698-3)
Supplement: Supplementary file 1 — Supplementary Figures. [file 41598_2021_88698_MOESM1_ESM.pdf]

In-depth transcriptomic analysis of human retina reveals molecular mechanisms underlying diabetic retinopathy

Kolja Becker[1], Holger Klein[1], Eric Simon[1], Coralie Viollet[1], Christian Haslinger[2], German Leparc[3], Christian Schultheis[3], Victor Chong[4], Markus H. Kuehn[5,6], Francesc Fernandez-Albert[1]*, Remko A. Bakker[7]*

Supplementary Figures S1-S6

Affiliations

[1]Global Computational Biology & Digital Sciences, Boehringer Ingelheim Pharma GmbH & Co. KG, Biberach an der Riß, Germany.

[2]Global Computational Biology & Digital Sciences, Boehringer Ingelheim RCV GmbH & Co KG, Vienna, Austria.

[3]Translational Medicine & Clinical Pharmacology, Boehringer Ingelheim Pharma GmbH & Co. KG, Biberach an der Riß, Germany.

[4]Therapeutic Area CNS Retinopathies Emerging Areas, BI International GmbH, Ingelheim, Germany.

[5]Department of Ophthalmology and Visual Sciences, University of Iowa, Iowa City, Iowa, United States.

[6]Center for the Prevention and Treatment of Visual Loss, Department of Veterans Affairs, Iowa City, IA 52246, USA

[7]Global Department Cardio-metabolic Diseases Research, Boehringer Ingelheim Pharma GmbH & Co. KG, Biberach an der Riß, Germany.

*Corresponding authors

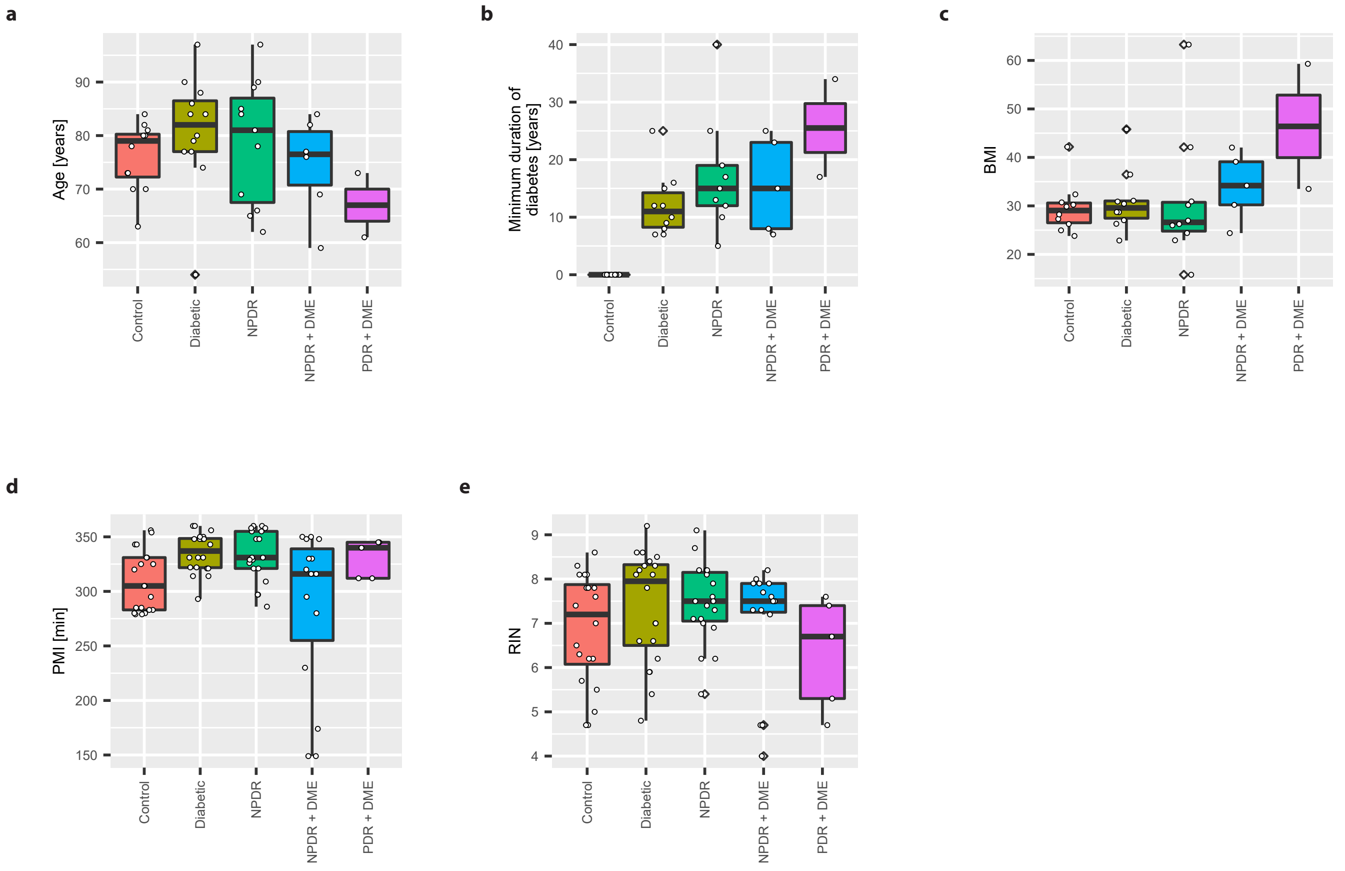

Figure S1 – Summary of per donor and per sample characteristics:

(a) Age distribution across donor groups. Black line denotes median age. Boxplot lower and upper hinges correspond to the first and third quartiles respectively. Whiskers extend to the largest expression value no further than 1.5 interquartile range from the hinge. Outliers are shown as white diamonds.

(b) Minimum duration of diabetes for each donor group. Boxplot details of Figure S1b apply. Missing values are indicated in Supplementary Table S1.

(c) Body Mass Index (BMI) for each donor group. Boxplot details of Figure S1b apply. Missing values are indicated in Supplementary Table S1.

(d) Per sample post-mortem intervals (PMI) from time of death until enucleation and sample fixation. Boxplot details of Figure S1b apply.

(e) Distribution of per sample RNA integrity number (RIN) across sample groups. Boxplot details of Figure S1b apply.

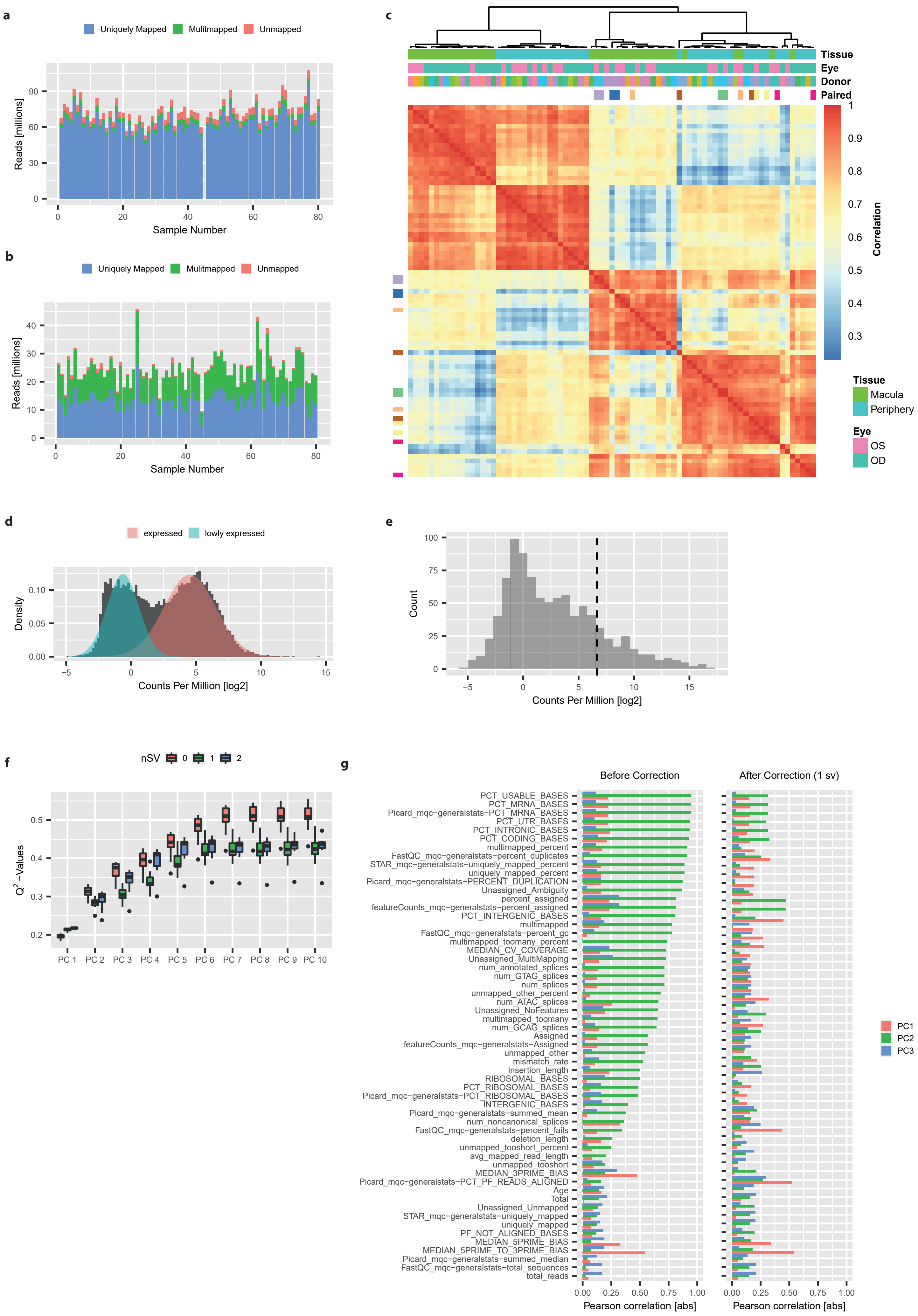

a
Uniquely Mapped
Mulitmapped
Unmapped
Reads [millions]
Sample Number
b
Uniquely Mapped
Mulitmapped
Unmapped
Reads [millions]
Sample Number
c
Tissue
Eye
Donor
Paired
Correlation
Tissue
Macula
Periphery
Eye
OS
OD
d
expressed
lowly expressed
Density
Counts Per Million [log2]
e
Count
Counts Per Million [log2]
f
nSV
Q2 −Values
PC 1
PC 2
PC 3
PC 4
PC 5
PC 6
PC 7
PC 8
PC 9
PC 10
g
Before Correction
After Correction (1 sv)
PCT_USABLE_BASES
PCT_MRNA_BASES
Picard_mqc−generalstats−PCT_MRNA_BASES
PCT_UTR_BASES
PCT_INTRONIC_BASES
PCT_CODING_BASES
multimapped_percent
FastQC_mqc−generalstats−percent_duplicates
STAR_mqc−generalstats−uniquely_mapped_percent
uniquely_mapped_percent
Picard_mqc−generalstats−PERCENT_DUPLICATION
Unassigned_Ambiguity
percent_assigned
featureCounts_mqc−generalstats−percent_assigned
PCT_INTERGENIC_BASES
multimapped
FastQC_mqc−generalstats−percent_gc
multimapped_toomany_percent
MEDIAN_CV_COVERAGE
Unassigned_MultiMapping
num_annotated_splices
num_GTAG_splices
num_splices
unmapped_other_percent
num_ATAC_splices
Unassigned_NoFeatures
multimapped_toomany
num_GCAG_splices
Assigned
featureCounts_mqc−generalstats−Assigned
unmapped_other
mismatch_rate
insertion_length
RIBOSOMAL_BASES
PCT_RIBOSOMAL_BASES
Picard_mqc−generalstats−PCT_RIBOSOMAL_BASES
INTERGENIC_BASES
Picard_mqc−generalstats−summed_mean
num_noncanonical_splices
FastQC_mqc−generalstats−percent_fails
deletion_length
unmapped_tooshort_percent
avg_mapped_read_length
unmapped_tooshort
MEDIAN_3PRIME_BIAS
Picard_mqc−generalstats−PCT_PF_READS_ALIGNED
Age
Total
Unassigned_Unmapped
STAR_mqc−generalstats−uniquely_mapped
uniquely_mapped
PF_NOT_ALIGNED_BASES
MEDIAN_5PRIME_BIAS
MEDIAN_5PRIME_TO_3PRIME_BIAS
Picard_mqc−generalstats−summed_median
FastQC_mqc−generalstats−total_sequences
total_reads
Pearson correlation [abs]
Pearson correlation [abs]
PC1
PC2
PC3

Figure S2 – Quality control and pre-processing:

(**a**) Uniquely mapped, multi-mapped, and unmapped read counts [million] of mRNA sequencing per sample. Sample 45 was excluded due to low total read count.

(**b**) Uniquely mapped, multi-mapped, and unmapped read counts [million] of miRNA sequencing per sample.

(**c**) Heatmap indicating Pearson correlation values between expression values [log2 CPM] for each sample. Dendogram represents hierarchical clustering of samples using a Pearson correlation distance metric and complete linkage grouping. Paired samples from the same donor and tissue combination, but different eyes, are indicated.

(**d**) Gaussian mixture model to identify non-expressed mRNA. Histogram denotes frequency of mean mRNA expression values [log2 CPM]. Distributions of the fitted Gaussian mixture model shown in blue (lowly expressed genes) and red (expressed genes).

(**e**) Histogram of miRNA expression values [log2 CPM]. Dashed line indicates the lower threshold (log2(100)) chosen for filtering active miRNA.

(**f**) Boxplot of cross-validated (5-fold) Q2-values of principal component analysis before confounder correction (nSV: 0) and after correction with 1 or 2 surrogate variables (nSV: 1, 2).

(**g**) Barplot of absolute Pearson correlation values between principal components (PC1-3) of the data and RNA sequencing metrics before and after confounder correction using 1 surrogate variable.

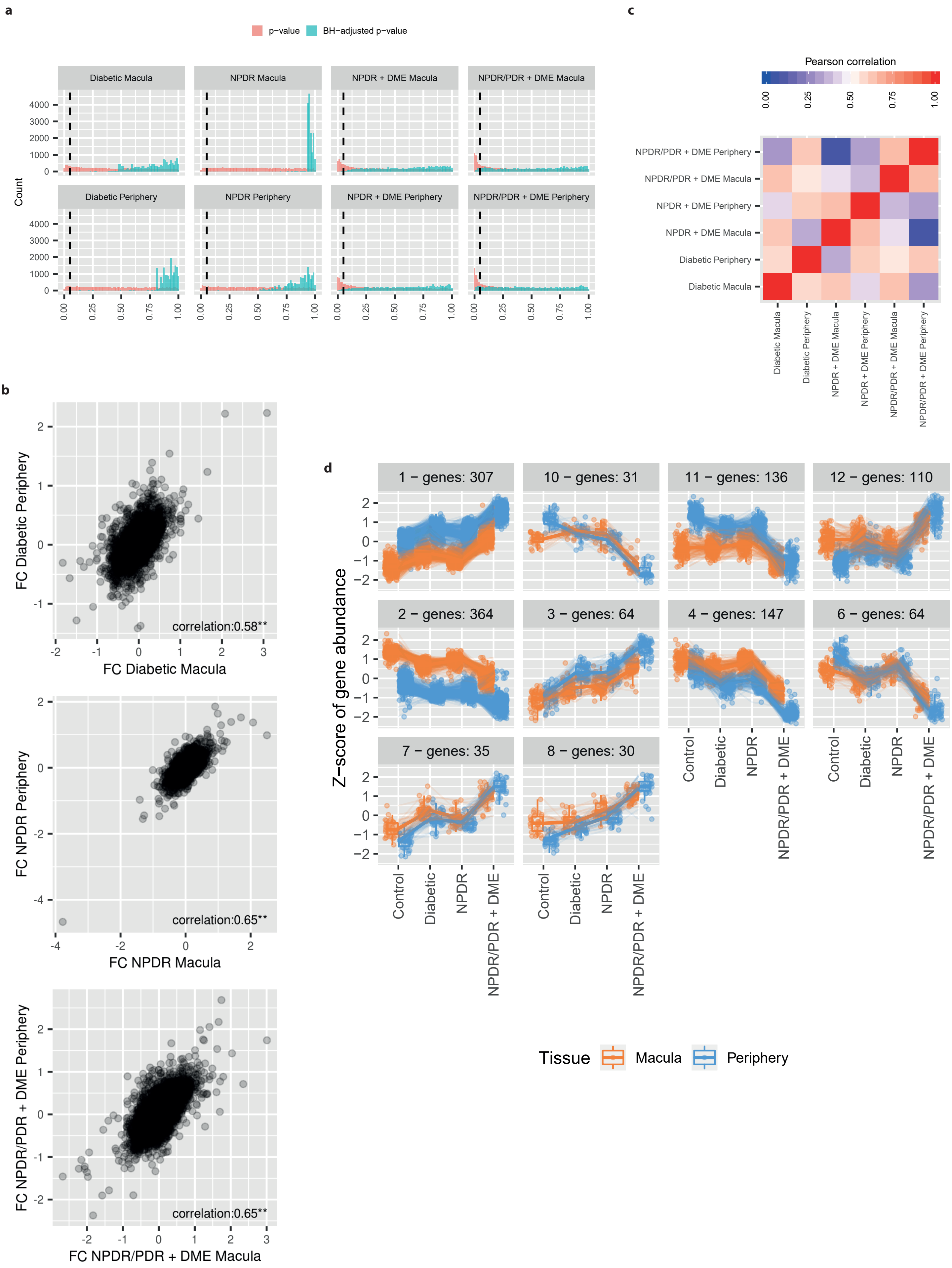
a
p−value
BH−adjusted p−value
Diabetic Macula
NPDR Macula
NPDR + DME Macula
NPDR/PDR + DME Macula
Diabetic Periphery
NPDR Periphery
NPDR + DME Periphery
NPDR/PDR + DME Periphery
Count
0
1000
2000
3000
4000
0.00
0.25
0.50
0.75
1.00
c
Pearson correlation
0.00
0.25
0.50
0.75
1.00
NPDR/PDR + DME Periphery
NPDR/PDR + DME Macula
NPDR + DME Periphery
NPDR + DME Macula
Diabetic Periphery
Diabetic Macula
b
FC Diabetic Periphery
FC Diabetic Macula
correlation:0.58**
FC NPDR Periphery
FC NPDR Macula
correlation:0.65**
FC NPDR/PDR + DME Periphery
FC NPDR/PDR + DME Macula
correlation:0.65**
d
1 − genes: 307
10 − genes: 31
11 − genes: 136
12 − genes: 110
2 − genes: 364
3 − genes: 64
4 − genes: 147
6 − genes: 64
7 − genes: 35
8 − genes: 30
Z−score of gene abundance
Control
Diabetic
NPDR
NPDR/PDR + DME
Tissue
Macula
Periphery

Figure S3 – Differential gene expression analysis:

(**a**) Distribution of p-values (red) and BH-corrected p-values (blue) from differential gene expression analysis. Threshold for significance (BH-adjusted p-value <0.05) is shown as dashed line.

(**b**) Heatmap of Pearson correlation values between fold-changes of each disease group vs healthy controls.

(**c**) Scatter plots comparing expression changes calculated from each sample group vs healthy controls between macula and periphery. Pearson correlation coefficient is given in the lower right corner. Double-asterisk denotes significance of correlation $p < 0.001$.

(**d**) Clustered expression values [z-score normalized log2 CPM values] of RNA identified as differentially regulated in NPDR/PDR + DME samples compared to healthy controls. For expression clustering, we made use of the degPatterns function provided by the DEGreport R package (see methods for details).

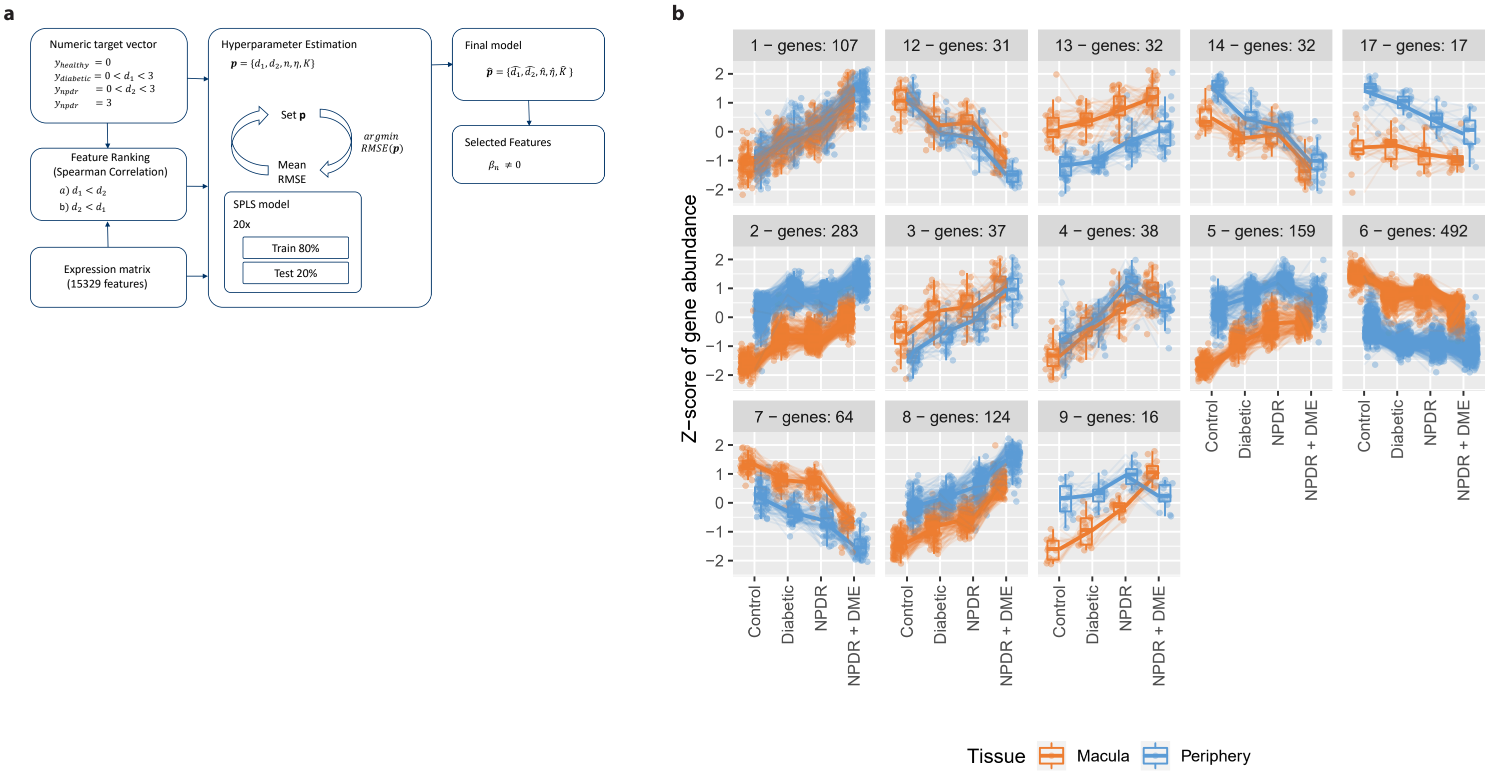

Figure S4 – Disease progression model:

(**a**) Schematic representation of the sparse partial least squares model to identify disease progression associated transcripts.

(**b**) Clustered expression values [z-score normalized log2 CPM values] of identified disease progression transcripts. For expression clustering, we made use of the degPatterns function provided by the DEGreport R package (see methods for details).

a

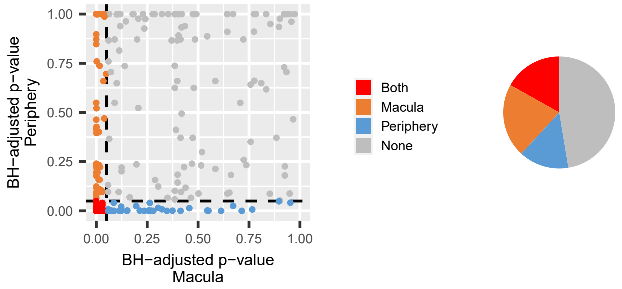

Figure S5 – Integration of miRNA and mRNA data:

(a) Comparison of BH-corrected p-values from Kolmogorov-Smirnov test. The Kolmogorov-Smirnov statistic tests for a negative skew in the distribution of correlation values between each miRNA and its putative targets. Resulting BH-adjusted p-values from this test are shown on the right panel. miRNA with significant (BH-adjusted p-value <0.05) reduced distribution in both sample sites (Macula and periphery) are colored red, while macula or periphery specific miRNA are shown in orange and blue respectively. Pie chart (right panel) indicates the fraction of miRNA in each class.

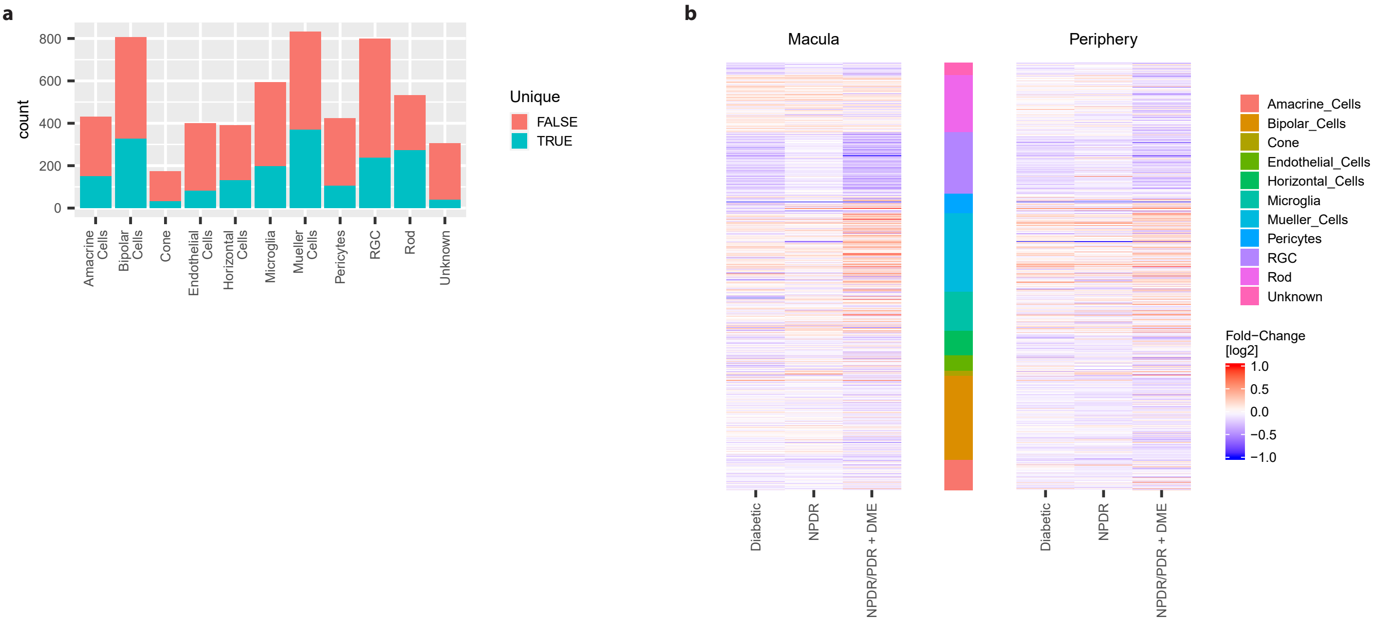

Figure S6 – Integration of single cell RNASeq Data:

(**a**) Barplot of cell type specific markers identified from human retina single cell RNA expression data. Shown are identified cell specific marker genes (Bonferroni-adjusted p-value < 0.01) for each retinal cell type. Genes specific to only one cell type are shown in blue, while genes associated with multiple cell types are shown in red.

(**b**) Heatmap showing log2 fold-changes of all cell type specific marker genes in defined disease groups vs healthy controls. Left panel corresponds to gene expression changes in macula samples, while right panel refers to gene expression changes observed in periphery samples.
